# Transannular bovine jugular vein monocusp for the reconstruction of severe right ventricular outflow tract obstruction

**DOI:** 10.1016/j.xjtc.2023.03.013

**Published:** 2023-04-05

**Authors:** Luigi Di Pasquale, Olivia Jaeger, Tugba Erdil, Martin Christmann, Daniel Quandt, Robert Cesnjevar, Hitendu Dave

**Affiliations:** a Division of Congenital Cardiovascular Surgery, Pediatric Heart Centre & Children's Research Centre, University Children’s Hospital Zürich, Zürich, Switzerland; b Division of Pediatric Cardiology, Kinderspital Luzern, Luzern, Switzerland; c Division of Cardiology, Pediatric Heart Centre & Children's Research Centre, University Children’s Hospital Zürich, Zürich, Switzerland

**Keywords:** tetralogy of Fallot, RVOT, transannular patch, monocusp, Contegra monocusp, pulmonary valve reconstruction, pulmonary annulus Z score

## Abstract

**Objective:**

Severe right ventricular outflow tract obstruction in tetralogy of Fallot and variants necessitates the use of transannular patch in a significant proportion of children undergoing repair. We have used a Contegra monocusp together with delamination of native leaflet tissue in order to create a functioning pulmonary valve.

**Methods:**

In total, 18 (2017-2022) consecutive Contegra monocusp implantations were included. Median age and weight were 3.65 [2.00; 9.43] months and 6.12 [4.30; 8.22] kg, respectively. Nine of 18 patients had undergone palliation. Native pulmonary leaflet tissue was recruited to create a single posterior cusp. Contegra monocusp selection was based on the goal to achieve a neoannulus of Z value ≈ 0. Monocusp sizes implanted were 16 [14; 18] mm. Patch plasty of left pulmonary artery (LPA) (9), right pulmonary artery (RPA) (2), and both LPA-RPA (5) were often performed.

**Results:**

All patients survived the operation and were discharged home in good health. Median ventilation time and hospital stay were 2 [1; 9] and 12.5 [9; 54] days, respectively. Follow-up duration was 30.68 [3.47; 60.47] months and 100% complete. One patient with well-corrected right ventricular outflow tract died 9.4 months postoperatively, possibly of aspiration. One child with membranous pulmonary atresia needed reoperation (conduit insertion) at 3.5 months of follow-up. Five needed catheter interventions: supravalvar stent (2), LPA stent (3), and RPA stent (1), most of them in the earlier half of the experience. Pulmonary annulus changed from preoperative –3.91 [–5.98; –2.23] to –0.10 [–1.44; 1.92] at discharge; growing proportionally to –0.13 [–3.52; 2.73] at follow-up. Kaplan–Meier freedom from composite dysfunction was 79.25 (95% confidence interval, +13.68%, –31.44%) at 36 months.

**Conclusions:**

Recruitment of native leaflets, optimal Contegra monocusp, and commissuroplasty provide an easily replicable technique for achieving a competent, proportionally growing neopulmonary valve. Longer follow-up is needed to determine its impact on delaying a pulmonary valve replacement.

**Video Abstract:**

## Graphical Abstract

**Figure:**
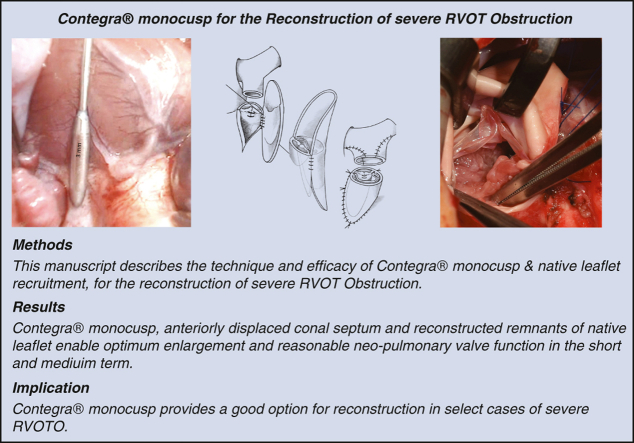

**Figure:**
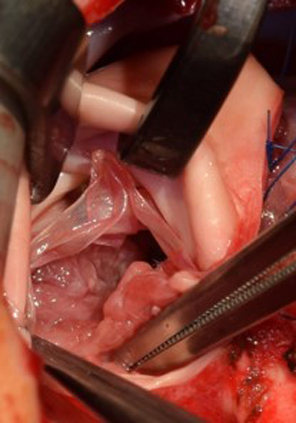
Contegra monocusp transannular patch.

**Central Message**

Native leaflet tissue recruitment, anteriorly displaced conal septum, and Contegra monocusp enable optimum enlargement and reasonably good competence of the neopulmonary valve.

**Perspective**

Transannular Contegra monocusp for reconstruction of complex RVOT Obstruction improves early outcome and may delay the need for reoperation.

The severity of the pulmonary annular hypoplasia, the number and quality of pulmonary leaflet tissue available, and also the adequacy of pulmonary valve function achieved after repair determine the long-term outcome after repair of tetralogy of Fallot (TOF).^1^ Despite restrictive strategies, a significant proportion of TOF repairs involve a transannular patch. Although the overall early mortality remains low,^1^^,^^2^ the pulmonary valve function influences the early- and long-term postoperative course.^3^, ^4^, ^5^

Various monocusp techniques using homograft,^6^ xenograft,^7^^,^^8^ polytetrafluoroethylene (PTFE; 0.1 mm),^5^^,^^9^ and Contegra monocusp^10^ have been described. We have used the Contegra monocusp with a goal to achieve a neopulmonary annular Z-value of approximately 0 and added pulmonary reconstructive techniques including delamination, native leaflet tissue recruitment, and optimization of Contegra free leaflet margin size by commissural plication to achieve pulmonary competence. The purpose of this study is to describe our technique and review the mid-term results of a using a Contegra monocusp transannular patch for right ventricular outflow tract (RVOT) reconstruction in children with severe forms of TOF and its variants.

## Methods

### Institutional Review Board Statement

The study design was conducted in accordance with the Declaration of Helsinki and approved by the Cantonal Ethics Committee of Zürich, Switzerland (BASEC protocol code no. 2017-01321) with an amendment dt. 11.11.2020. Informed consent was obtained from the patients’ parents.

### Patients

Eighteen consecutive Contegra monocusp transannular patch insertions performed during repair of severe forms of TOF and its variants by a single surgeon (H.D.) (2017-2022) were included in the study. All patients received postoperative aspirin. Demographic, clinical, operative, and follow-up data were retrospectively collected from digital hospital database as well as from external referring physicians (Table 1).

**Table 1:** Demographics and preoperative features

N (%)	18 (100)
Male sex, n (%)	10 (55.56)
Age, median [min; max]	
Age at palliation, d	6.00 [5.00; 18.00]
Age at operation, mo	3.65 [2.00; 9.43]
Duration since palliation, mo	4.00 [2.30; 9.23]
Body profile, median [min; max]	
Weight, kg	6.12 [4.30; 8.22]
Height, cm	60.25 [53.00; 71.00]
Body surface area, m^2^	0.32 [0.23; 0.37]
Native pathology, n (%)	
TOF	10 (55.56)
DORV Fallot type	3 (16.67)
Membranous pulmonary atresia Fallot type	4 (22.22)
AVSD Fallot type	1 (5.56)
RVOTO substrate, n (%)	
Infundibular	18 (100)
Membranous valvular atresia	4 (22.22)
Valvular dysplasia	8 (44.44)
Valvular and supravalvar hypoplasia	18 (100)
LPA hypoplasia	15 (83.33)
RPA hypoplasia	10 (55.56)
LPA isolation (perfusion via atypical PDA)	1 (5.56)
Previous palliations (9 patients), n (%)	
Central shunt	5 (27.78)
PDA stent	2 (11.11)
RVOT stent	2 (11.11)
MAPCAs closure	2 (11.11)
PDA ligature	2 (11.11)
LPA patch plasty	1 (5.56)
Additional cardiac diagnosis, n (%)	
ASD II/PFO	18 (100)
ASD I	2 (11.11)
PDA	5 (27.78)
AVSD common valve, ASD I	1 (5.56)
MAPCAs	3 (16.67)
Right aortic arch	5 (27.78)
Bicuspid aortic valve	2 (11.11)
Left SVC draining into coronary sinus	1 (5.56)

*TOF*, Tetralogy of Fallot; *DORV*, double-outlet right ventricle; *AVSD*, atrioventricular septal defect; *RVOTO*, right ventricular outflow tract obstruction; *LPA*, left pulmonary artery; *RPA*, right pulmonary artery; *PDA*, patent ductus arteriosus; *RVOT*, right ventricular outflow tract; *MAPCA*, major aortopulmonary collateral arteries; *ASD*, atrial septal defect: *PFO*, patent foramen ovale; *SVC*, superior vena cava.

### Decision-Making

Based on preoperative echo and intraoperative Z-score measurements,^11^ the following algorithm guided the decision-making approach to the pulmonary annulus:

Z score approximately ≥−2: provided the pulmonary leaflets were normally laid with bi/tricuspid configuration, an annulus-sparing approach was preferred.

Z score −2 to −3: If the pulmonary valve was pliable, a trial with annulus-sparing approach, aggressive commissurotomy, and intraoperative balloon dilatation was undertaken. If the postrepair RVOT z score was <−2 and the right ventricle-to-pulmonary artery gradient was >20 to 25 mm Hg (mean Doppler gradient) with right ventricular/left ventricular pressure ratio of >0.65 to 7, a transannular approach was justified. In case of transversely oriented pulmonary valve with 2 well-formed cusps and a small annulus, a pulmonary valve cusp augmentation with autologous pericardium as proposed by Sung and colleagues was preferred.^3^^,^^12^

Z score <−3: Extreme pulmonary annular hypoplasia with dysplastic, nonpliable and nodular leaflets necessitated a transannular monocusp reconstruction.

### Chronology of Surgical Steps

**Figure 1:**
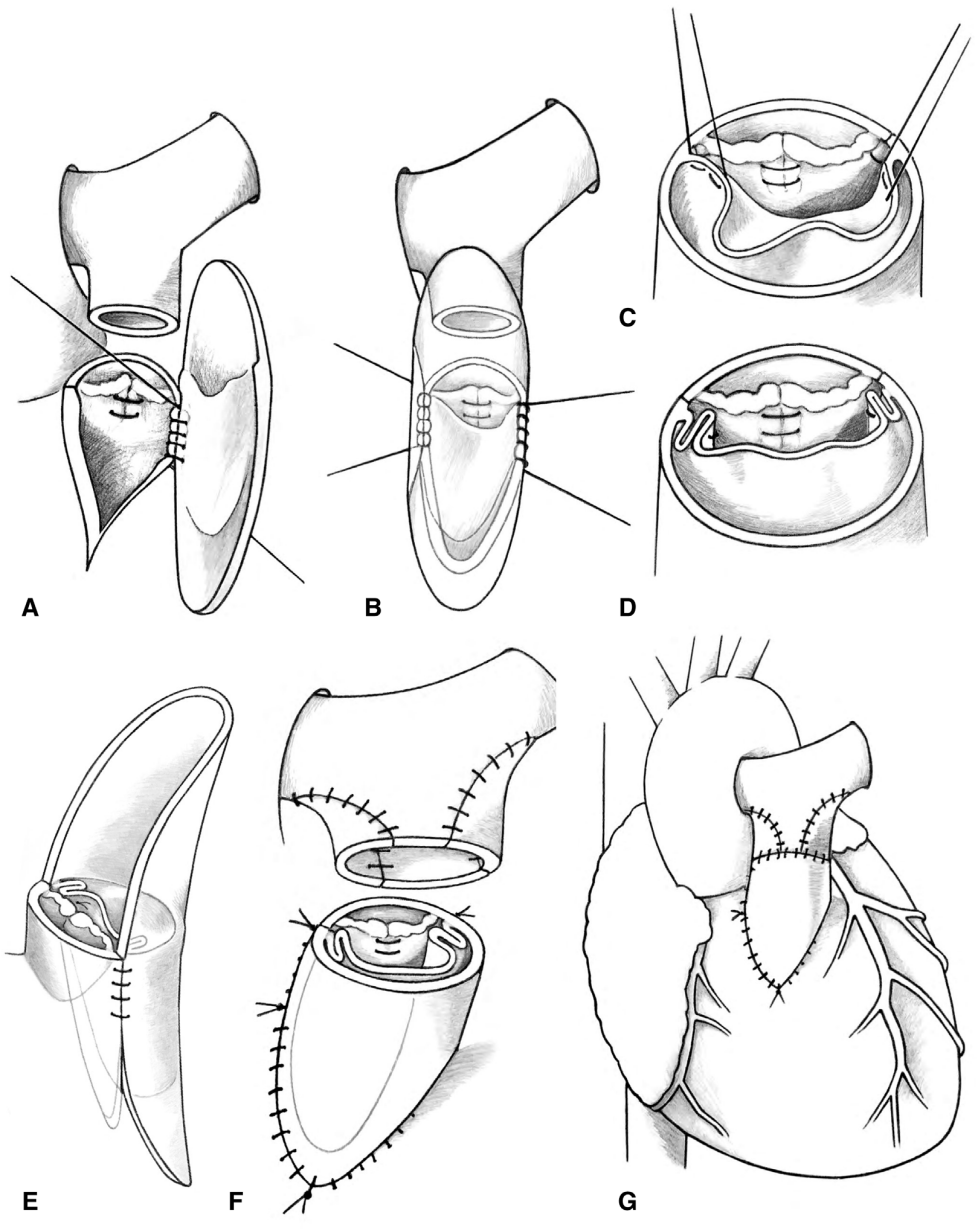
A and B, Beginning suturing Contegra monocusp at the left-sided commissure and running downwards. Once halfway, the right-sided suture-line is initiated at the *upper* end of the commissure running *downwards*. The proximal lip of the suture line is completed after trimming the Contegra. C-E, Commissural plication ensures native-Contegra leaflet apposition; at the same time shortening the free edge of the leaflet. F, Triangular patch enlargement of either the proximal LPA or proximal LPA + RPA to enlarge the bifurcation commensurate enough to match the enlarged pulmonary root. G, Completed procedure. *LPA*, Left pulmonary artery; *RPA*, right pulmonary artery.

Figure 1, *A-F* shows the schematic representation of our technique of Contegra monocusp implantation. After establishing moderately hypothermic cardiopulmonary bypass, the ductal ligament is divided and the pulmonary tree is extensively mobilized. Upper infundibulotomy is performed on a cardioplegic heart and accessory infundibular muscles are transected (often with electrocautery to prevent coronary fistula). The pulmonary valve is inspected and sized using olive tip probes. For a severely hypoplastic pulmonary trunk with dysplastic pulmonary valve, a Contegra monocusp transannular patch is chosen.

### Choosing Contegra Monocusp Size

Considering that a child needs a 10-mm pulmonary annulus, and his native annulus is 4 mm, the size of the Contegra monocusp chosen is calculated as follows:

$${TAP}_{contegra}=\pi \;\left(expected\;diameter-native\;diameter\right)$$

=3.14(10−4)

=18.8mm

The appropriately sized Contegra is ordered for saline irrigation. During this time, ventricular septal defect closure is performed.

### Delamination and Creating a Dorsal Leaflet

Native pulmonary leaflet tissue is analyzed. Using a combination of take-down of the commissure, delamination, fusion of leaflets, and leaflet thinning, a dorsal neopulmonary leaflet is created (Figure 1, *A*).

### Neopulmonary Root Creation

Contegra monocusp is sutured beginning at the left-sided commissure with the monocusp lying on its back, followed by suturing of the right-sided margin (Figure 1, *A-C*). Thereafter, the proximal tongue is fashioned so as to match the ventriculotomy. Due to excessive height of the leaflets, monocusp commissures need to be higher than the native leaflet commissures in order to ensure Contegra free leaflet edge to the native leaflet apposition.

### Commissural Plasty

**Figure 2:**
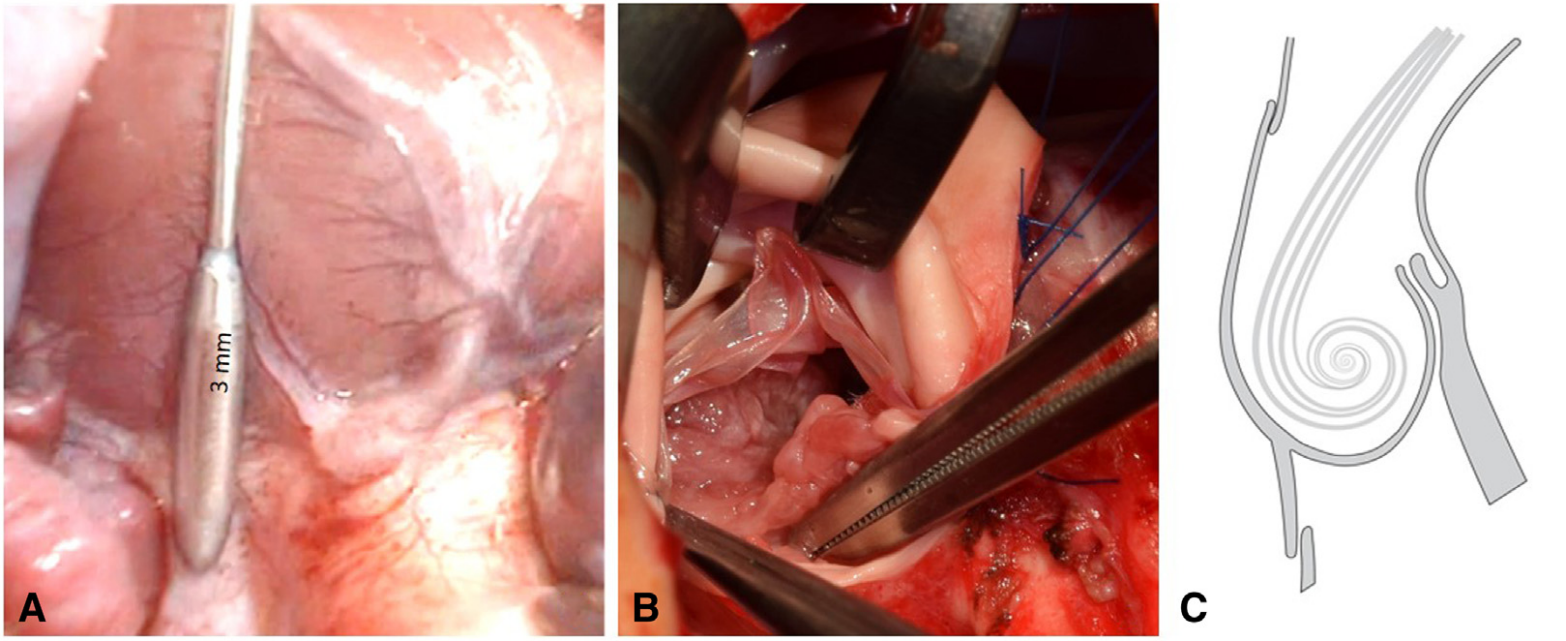
A, Extremely hypoplastic Pulmonary annulus and the main pulmonary artery; B, Intraoperative photograph of the neopulmonary valve: Anterior two-thirds contributed by the Contegra monocusp and posterior one-third by the native pulmonary valvar tissue. C, The eddy currents as hypothesized to be working in diastole to close the monocusp.

In our experience, for a given Contegra monocusp size, the corresponding free leaflet margin is too redundant to be competent. Hence, commissural plication of the monocusp to the native leaflet commissure is undertaken in order to reduce its redundancy (Figure 1, *D* and *E*). A harmonic coaptation at the commissures and not-too much redundancy of the monocusp are key to achieving valve competence (Figure 2, *A*).

### Enlargement of Pulmonary Annulus (PA) Bifurcation

The PA bifurcation may be enlarged by separate patches on the left PA or right PA or both to match the size of the neopulmonary root. Creating an enlarged bifurcation to match the enlarged root is equally critical. We have used different autologous and xenogenic pericardial patches (Table 2) for branch PA enlargement but have preferred spongiosa-free Contegra patches lately due to their elasticity, hemostatic nature, and smooth inner wall.

**Table 2:** Intraoperative findings

RVOT procedure	n (%)
Contegra, mm used for monocusp, median [min; max]	16 [14; 18]
No. of 14-mm Contegra used	2 (11.11)
No. of 16-mm Contegra used	8 (44.44)
No. of 18-mm Contegra used	8 (44.44)
Pulmonary tree augmentation: patch-plasty	16 of 18 patients (88.89)
LPA	9 (50.00)
RPA	2 (11.11)
Both LPA/RPA	5 (27.78)
Posterior bifurcation plasty	2 (11.11)
Unifocalization LPA-RPA	1 (5.56)
Type of patch material	
Autologous untreated pericardium	3 (16.67)
Equine heterologous pericardium	2 (11.11)
Bovine heterologous pericardium	6 (33.33)
Contegra patch (spongiosa-free)	5 (27.78)
Pulmonary valve plasty	12 (66.67)
Fusion of leaflet	3 (25.00)
Takedown of commissure and delamination	6 (50.00)
Leaflet thinning	1 (08.33)
Not reported	4 (33.33)

Intracardiac procedures description	n (%)
ASD closure	18 (100)
VSD closure	18 (100)
RVOT muscle bundles resection	18 (100)
AVSD repair (double-patch technique); MVR, TVR	1 (5.56)
Tricuspid valvuloplasty	4 (22.22)
Shunt take-down	5 (27.78)
PDA stent removal	2 (11.11)
RVOT stent removal	2 (11.11)
MAPCAs closure	1 (5.56)
Intraoperative pRV:LV, median [min; max]	0.56 [0.39; 0.70]
Cardiopulmonary bypass, median [min; max]	
CPB time, min	240 [189; 463]
Crossclamp time, min	147 [100; 250]

*RVOT*, Right ventricular outflow tract; *LPA*, left pulmonary artery; *RPA*, right pulmonary artery; *ASD*, atrial septal defect; *VSD*, ventricular septal defect; *AVSD*, atrioventricular septal defect; *MVR*, mitral valve repair; *TVR*, tricuspid valve repair; *PDA*, patent ductus arteriosus; *MAPCA*, major aortopulmonary collateral arteries; *pRV:LV*, right ventricular/left ventricular pressure ratio; *CPB*, cardiopulmonary bypass.

### Intraoperative Findings

Intraoperative findings are summarized in Table 2.

### Echocardiography: Pitfall

Although the measurement of RVOT gradient can be performed conventionally by continuous-wave Doppler, the estimation of neopulmonary valve regurgitation must take into consideration the fact that the Contegra monocusp is displaced caudally to the junction of right ventricular (RV) body to the RV infundibulum. This addition of contractile part to the neopulmonary trunk may cause a reversal of flow in pulmonary side branches, even when no significant regurgitation is present. Hence, corroborative evidence with color Doppler estimation of the vena contracta and the extent of regurgitation into RV cavity is mandatory. The observed regurgitation was graded as grade 1 to 3 for mild, moderate, and severe.^13^

### Statistical Analysis

Data are presented as median [range]. Survival was determined using Kaplan–Meier curves. Z scores were calculated according to Pettersen and colleagues.^11^ Statistical analysis was performed using Microsoft Excel 2019 and GraphPad Prism 9.5.0.

## Results

All children survived the operation. The median follow-up duration was 100% complete at 30.68 [3.47-60.47] months.

### Mortality

One child in good clinical and cardiac status died at home while breast feeding (9.4 months postoperatively), probably due to aspiration. No autopsy was performed. An echocardiography 3 weeks before death showed a mean gradient of 18 mm Hg and a mild pulmonary regurgitation (PR). No arrhythmias were reported. Kaplan–Meier survival of our cohort was 93.3% (95% confidence interval [CI], +5.7, –32.1) at 36 months.

### Reoperation

One child with pulmonary atresia ventricular septal defect (case no. 8, performed in 2018) needed reoperation with implantation of a Contegra valved conduit for residual pulmonary artery stenosis at 3.5 months after total correction. A freestanding Contegra leaflet implanted posteriorly in addition to the Contegra monocusp was in retrospect an error. All 16 remaining children are alive, thriving, and free of reoperation. Freedom from reoperation is 94.4% (95% CI, +4.8, –27.8) at 36 months.

### Catheter Reintervention

Five children required catheter reintervention on the pulmonary tree after a median time of 10.30 months. Freedom from reintervention is 66% (95% CI, +18.5, −30.1) at 36 months. Details about reintervention are depicted in Table 3.

**Table 3:** Outcomes

Ventilation duration, d	2 [1; 9]
Intensive care unit stay, d	5.5 [2; 13]
Hospital stay, d	12.5 [9; 54]
Early mortality (30-d in hospital), n (%)	0 (0)
Late mortality, n (%) (exitus 9.43 mo after operation)	1 (5.56)
Follow-up duration, mo	30.68 [3.47; 60.47]
Postoperative complications, n (%)	
Pleural effusion	1 (5.56)
Transient arrhythmia	4 (22.22)
Transient renal impairment (requiring PD)	2 (11.11)
Reoperation, n (%)	
RV-PA Contegra conduit 12 mm (technical error)	1/18 (5.56)
Time to reoperation, mo	3.5
Kaplan–Meier freedom from reoperation, % at 36 mo, % (95% CI)	94.4 (+ 4.8, –27.8)
Reintervention on pulmonary tree, n (%)	5/18 (27.8%)
Stent MPA (without encroaching on the monocusp)	2 (11.11)
Stent RPA	1 (5.56)
Stent LPA	3 (16.67)
Balloon angioplasty	2 (11.11)
Time to reintervention, mo	10.30 [2.60; 30.43]
Kaplan–Meier freedom from catheter reintervention, % at 36 mo	66 (95% CI, + 18.5, −30.1)
Freedom from all reinterventions (catheter + reoperation), % (95% CI)	
Freedom from all reinterventions on PA trunk, % at 36 mo	79.7 (+13.4, −31.0)
Freedom from all reinterventions on -branch PA, % at 36 mo	74.2 (+15.3, −29.4)

All values are described as median [Range], except mentioned otherwise. *PD*, Peritoneal dialysis; *RV-PA*, right ventricle-to-pulmonary artery; *CI*, confidence interval; *MPA*, main pulmonary artery; *RPA*, right pulmonary artery; *LPA*, left pulmonary artery; *PA*, pulmonary annulus.

### Catheter Plus Surgical Reinterventions

**Figure 3:**
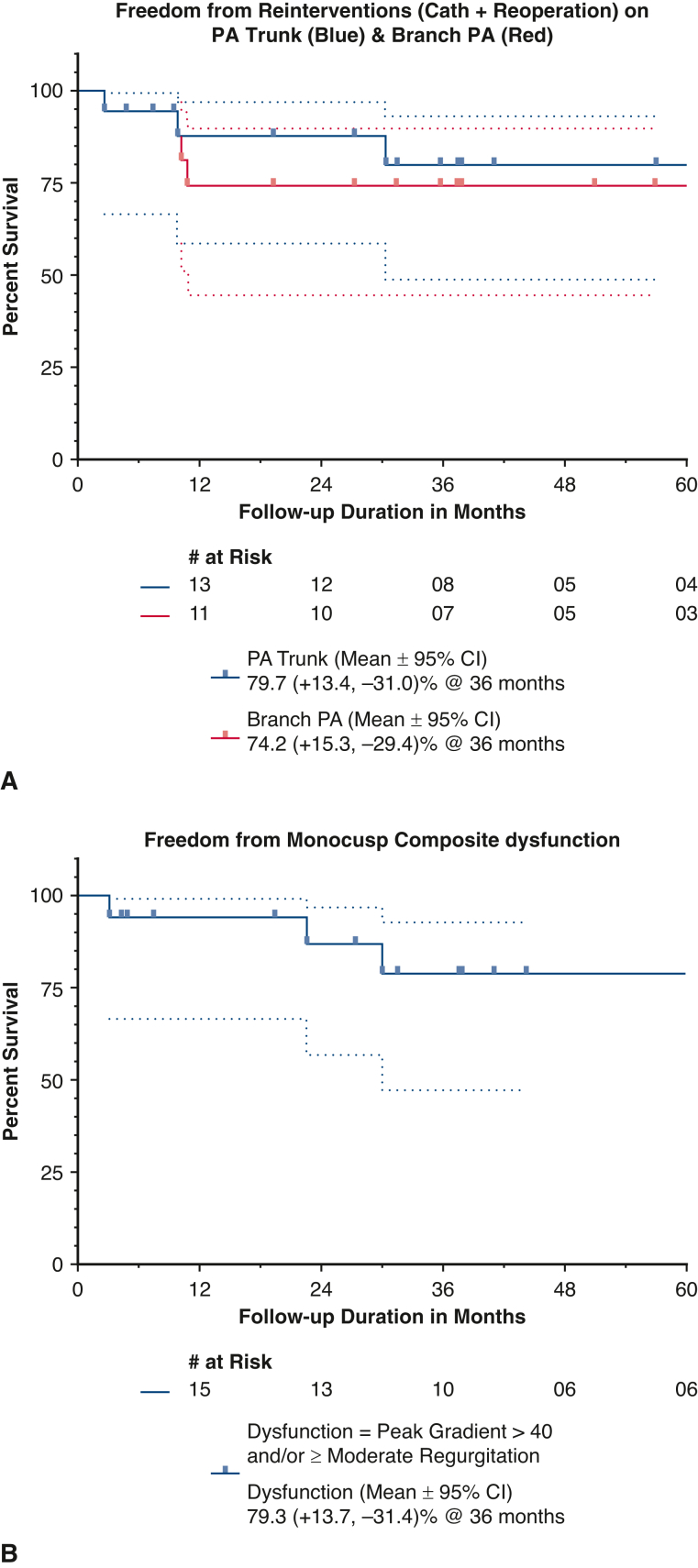
A, Kaplan–Meier freedom from all reinterventions (catheter + reoperation) on PA trunk (*blue*) and on branch PA (*red*). B, Kaplan–Meier freedom from composite dysfunction. *PA*, Pulmonary artery.

When considering all adverse events (catheter plus surgical reinterventions), the freedom from same on pulmonary trunk and on-branch PA was 79.7% (+13.4, −31.0) and 74.2% (+15.3, −29.4) at 36 months’ respectively (Figure 3, *A*).

### Neopulmonary Valve Growth and Function

Pulmonary annulus changed from preoperative –3.91 [–5.98; –2.23] to –0.10 [–1.44; 1.92] at discharge; growing proportionally to –0.13 [–3.52; 2.73] at follow-up. The mean Doppler gradient across the RVOT was 8.3 mm Hg [2.3-16.5] at discharge and increased to 11.6 mm Hg [3.6-30] at last follow-up. Median predischarge pulmonary regurgitation was grade 1 in 16 of 18 (88.88%) and grade 2 in 2 of 18 (11.11%). This changed to grade 1/1.5 in 14 of 16 (87.5%), grade 2 in 1 of 16 (6.25%), and grade 3 in 1 of 16 (6.25%) (excluding 1 mortality and 1 reoperation) at follow-up. When we defined composite dysfunction as peak gradient >40 mm Hg and/or moderate pulmonary regurgitation or greater, Kaplan–Meier freedom from dysfunction was 79.25% (95% CI, +13.68, –31.44) at 36 months (Figure 3, *B*). Pulmonary arterial growth is depicted in Table 4.

**Table 4:** Timeline: echo

Variable	Preoperative	Postoperative	Follow-up
Age, mo	3.65 [2.00; 9.43]	4.42 [2.73; 9.80]	34.90 [6.13; 66.43]
Follow-up duration, mo		0.38 [0.27; 1.73]	30.68 [3.47; 60.47]
Body profile, median [min; max]			
Weight, kg	6.12 [4.30; 8.22]	6.05 [4.12; 7.50]	13.95 [5.88; 20.75]
Height, cm	60.25 [53.00; 71.00]	61.50 [53.00; 71.00]	94.50 [61.00; 117.00]
Body surface area, m^2^	0.32 [0.23; 0.37]	0.30 [0.22; 0.36]	0.60 [0.30; 0.82]
Echocardiographic assessment (n = 17)			
Pulmonary annulus, Z value	−3.91 [−5.98; −2.23]	−0.10 [−1.44; 1.92]	−0.13 [−3.52; 2.73]
RPA, Z value	−1.67 [−4.30; 0.44]	−0.29 [−2.34; 2.05]	−0.75 [−3.98; 1.18]
LPA, Z value	−1.90 [−4.48; 0.99]	−0.49 [−1.95; 4.05]	−0.96 [−2.61; 0.83]
No. of patients with moderate PR or greater	0	2/18	2/16 (surviving monocusps)
Median PR (grade 0-3)		1.00 [0.50; 2.00]	1.00 [0.50; 3.00]
Mean gradient pulmonary valve, mm Hg		8.30 [2.30; 16.50]	11.60 [3.60; 30.00]
RV pressure estimation by TR jet, mm Hg		28 [14.00; 45.00]	29.50 [12.00; 45.00]

All values are described as median [range] except mentioned otherwise. *RPA*, Right pulmonary artery; *LPA*, left pulmonary artery; *PR*, pulmonary regurgitation; *RV*, right ventricular; *TR*, tricuspid regurgitation.

## Discussion

Since transannular patching remains a necessity, postoperative acute PR manifests as inotropic dependence, prolonged ventilation, and intensive care unit and hospital stay.^3^^,^^5^ Although long-term consequences of chronic PR continue to be debated, there is increasing consensus that it (in tandem with other operative insults such as myocardial scars) negatively impacts long-term outcome.^14^, ^15^, ^16^ Hence, various pulmonary valve reconstructive techniques have been used, to avoid extremes of gradient and regurgitation. The lesser the amount of native tissue available, the greater is the amount of foreign tissue necessary to create a functioning neopulmonary valve. For severely hypoplastic annulus with a dysplastic valve, an anterior monocusp is preferred to a conduit in infancy for obvious reasons.

### Homograft Monocusp

Although the use of homograft monocusp dates back to 1968,^7^^,^^8^ the results have been inconsistent, partly because it was only used as an onlay patch without resorting to significant reconstructive techniques.^6^^,^^8^^,^^9^ Nath and colleagues^9^ reported their experience of 131 pulmonary homograft monocusp implantations and showed good early outcome (ventilation and hospital stay of 1 and 6.5 days, respectively). Early mortality was 2.3% and late mortality was 4%. At a median follow-up of 5 years, 73% monocusp suffered from moderate or severe regurgitation. Freedom from pulmonary valve replacement was 85 ± 10.3% at 10 years.

### PTFE Monocusp

Turrentine and Brown popularized^5^^,^^17^ the PTFE (0.1 mm) monocusp and reported improved perioperative outcome in terms of duration of mechanical ventilation, intensive care unit, and hospital stay, even when 80.8% suffered moderate or severe regurgitation and 24.5% needed replacement at a follow-up of 10.9 ± 5.8 years. This procedure requires precise measurements of the monocusp and the overlying patch so as to achieve reasonable competence. This makes the outcome operator dependent and maybe difficult for surgeons in their learning curve.

Miyazaki and colleagues^18^ reported on a multi-institutional Japanese experience, with a PTFE valved patch and bulging sinuses in 534 patients. They reported excellent 10-year freedom from reoperation of 93% and freedom from moderate or severe regurgitation of 78% at a median follow-up of 4.6 years. Our present experience closely replicates the Japanese results.

### Other Monocusp Techniques

Monocusp created from autologous or xeno-pericardium,^4^ Dacron, and right atrial appendage have also been described with varying short- and long-term results.

### Contegra Monocusp

Chiappini and colleagues^10^ first reported use of Contegra monocusp as a transannular patch. They reported good outcomes, with approximately 36.4% showing moderate or severe PR at 28 months’ follow-up. Contegra enjoys long-term familiarity to surgeons with excellent reported outcomes.^19^ The multiple sizes, off-the-shelf availability, naturally defined relationship between the cusp and the sinus, greater height, increased leaflet coaptation, and adequate tissue proximal and distal to the valve makes it attractive for use as a monocusp, notwithstanding its cost. The Contegra valve leaflets are the more pliable of all xenogeneic tissues with low inertia, which means that they are seldom obstructive even if oversized and need little energy to open up from closed position. Worldwide experience with this concept is limited.^10^^,^^20^

We have built on the concept of Contegra monocusp, by proposing a simple technique based on following pillars:

1. The addition of deficient circumference by the Contegra monocusp with a goal of achieving a Z value ≈ 0. The monocusp thus presented possesses excessive free leaflet margin, which needs to be shortened, by using commissural plication between the Contegra and the native leaflet.
2. The larger coaptation area between the redundant leaflet and the anteriorly displaced conal septum may continue to provide competence with patient growth in the medium term (Figure 4).

**Figure 4:**
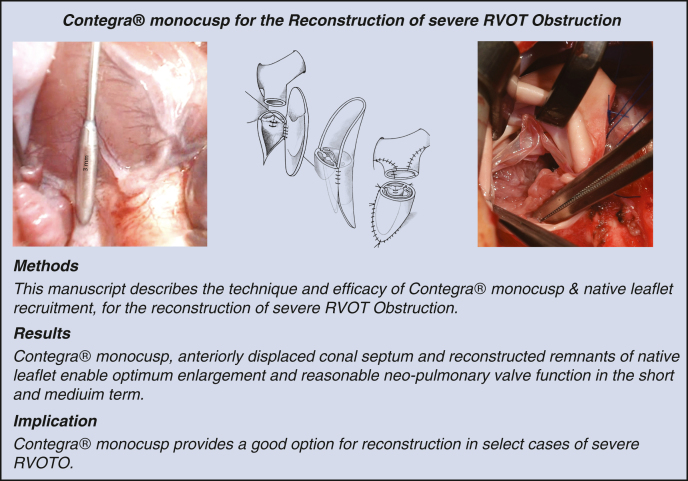
Graphic abstract of Contegra monocusp technique. *RVOT*, Right ventricular outflow tract.
3. The better the quality of native leaflet tissue and better the coaptation to the Contegra monocusp, the better the stimulus for the native leaflet to grow.
4. Time will tell whether this delayed progression of significant regurgitation helps delay the need for a pulmonary valve replacement.

### Flow Dynamics of Contegra Monocusp

The anterocephalic displacement of the conal septum in TOF creates an angle of approximately 45° between the RVOT and the pulmonary bifurcation. Furthermore, since the monocusp together with its sinus mimics the anatomy of an aortic sinus, it may be presumed that similar da Vinci vortices^21^ are at play in the Contegra monocusp (Figure 2, *B*). These eddy currents on the one hand distend the sinus outwards and on the other hand flap the leaflet dorsally. Even when no significant native leaflet tissue is available, the conal septal shelf provides a cushion for the monocusp to fall back upon and create pulmonary competence. Considering the severe RVOT obstruction with little native pulmonary tissue in our cohort, a duration of mechanical ventilation and hospital stay of 2 and 12.5 days was gratifying. Although the neopulmonary annulus grew commensurate with the child’s growth, the valve has continued to function very well in 14 of 16 surviving monocusps (excluding 1 patient who died and 1 who needed replacement). We believe that the good early outcome and the hope of better mid-term outcome justify the costs involved and the sacrifice of a complete Contegra conduit. However, all the hypothesis about the long-term outcome needs to be validated with longer follow-up in a larger cohort of patients.

### Contegra Patches for Branch PA Plasty

We have used different patch materials (autologous and xenogeneic) for branch PA reconstruction (Table 2). However, of late, we have relied on spongiosa denuded Contegra patches. While being guarded about its long-term impact, we have 5 reinterventions in autologous pericardial plasty (2) and xenopericardial plasty (3) and none in the group with Contegra patches (5).

### Future Outlook

Although the advantages of having a competent pulmonary valve have been well documented, its impact on long-term survival is unknown. Despite this, the general logic in favor of creating a functioning pulmonary valve justifies the quest for developing products and strategies that allow off-the-shelf, reasonably priced, and easy-to-learn concepts, which would help push behind the need for a complete valve by a decade or two. This in turn should allow putting in an adult-sized valve and thus better valve survival. These endeavors would go a long way in improving the morbidity and mortality of these complex patients. Democratization would necessitate translating this concept to a synthetic construct and industrializing it. The PTFE monocusp described by some Japanese groups^18^^,^^22^^,^^23^ has achieved excellent results; unfortunately, however, they remain unavailable for worldwide dissemination.

### Limitations

This retrospective analysis of our technique has inherent limitations of a single-institutional, retrospective observational study. The decision to go transannular is not based on any one objective criteria but involves subjectivity of the operating surgeon. Although echocardiography is the obvious diagnostic modality, the extra-anatomic position of the monocusp subjects it to fallacies in interpretation if standard criteria of regurgitation are used. Last but not the least, the long-term outcome of this approach is still a matter of conjecture.

## Conclusions

Contegra monocusp provides a simple, easily available transannular monocusp patch that helps reconstruct a reasonably functioning neopulmonary valve in severe form of TOF and variants. The neopulmonary annulus continues to grow proportionately in the medium term while maintaining competence, thanks to a redundant Contegra leaflet and the anteriorly displaced conal septum abutting each other. Despite the high material cost of the monocusp, the impact of smooth early postoperative course and a prospect of delayed need for a pulmonary valve makes it an attractive option. Apart from documenting the long-term outcome of our approach, future efforts should be directed at developing similar prosthetic constructs leading to industrialization and easy availability (Video Abstract).

### Conflict of Interest Statement

The authors reported no conflicts of interest.

The *Journal* policy requires editors and reviewers to disclose conflicts of interest and to decline handling or reviewing manuscripts for which they may have a conflict of interest. The editors and reviewers of this article have no conflicts of interest.

## Supplementary Data

Video Abstract Transannular bovine jugular vein monocusp for the reconstruction of severe right ventricular outflow tract obstruction: Technique and outcome. Video available at: https://www.jtcvs.org/article/S2666-2507(23)00112-8/fulltext.
